# 14-3-3σ is an independent prognostic biomarker for gastric cancer and is associated with apoptosis and proliferation in gastric cancer

**DOI:** 10.3892/ol.2014.2676

**Published:** 2014-11-06

**Authors:** YI-LIANG LI, LIHUA LIU, YANG XIAO, TAO ZENG, CHAO ZENG

**Affiliations:** 1 Department of Neurology, The Central Hospital of Loudi Affiliated to the University of South China, Loudi, Hunan 417000, P.R. China; 2 Department of Respiratory Disease, The First Affiliated Hospital of Guangxi Medical University, Nanning, Guangxi 530021, P.R. China; 3 Department of Orthopaedics, The Central Hospital of Loudi Affiliated to the University of South China, Loudi, Hunan 417000, P.R. China; 4 School of Laboratory Medicine, Guangdong Medical College, Dongguan, Guangdong 523808, P.R. China; 5 Department of Pathology, Guangdong Medical College, Dongguan, Guangdong 523808, P.R. China

**Keywords:** 14-3-3σ, prognosis, apoptosis, proliferation, immunohistochemistry

## Abstract

14-3-3 proteins participate in various cellular processes, including apoptosis, proliferation and malignant transformation. 14-3-3σ, a member of the 14-3-3 protein family, is important in several types of cancer; however, little is known about the clinical significance and biological roles of 14-3-3σ in gastric cancer. The present study analyzed the expression pattern of 14-3-3σ in gastric cancer and investigated its correlation with the prognosis of gastric cancer patients. Furthermore, the association of 14-3-3σ with Ki-67, Bcl-2 and Bax was evaluated. 14-3-3σ was expressed at higher level in gastric cancer tissue compared with healthy gastric tissue, and 14-3-3σ expression was significantly correlated with tumor size and tumor node metastasis stage (P<0.05). To the best of our knowledge, the present study data are the first to suggest that 14-3-3σ expression has been significantly associated with poor prognosis in gastric cancer. Additionally, 14-3-3σ overexpression was positively correlated with Ki-67 and Bcl-2 expression levels. Thus, 14-3-3σ is a potential prognostic marker for gastric cancer patients, and may be involved in regulating the apoptosis and proliferation of gastric cancer cells.

## Introduction

Despite the declining incidence of gastric cancer in certain parts of the world, it remains one of the leading causes of cancer-related mortality worldwide (1). Gastric cancer is hypothesized to develop in a multi-step process that includes the activation and overexpression of oncogenes, such as K-sam and c-Met (2,3), as well as the inactivation of tumor suppressor genes, such as APC (4). Various genes are associated with stomach cancer, such as TP53 (5), TGF-β (6) and Runx3 (7); however, their association with gastric cancer is weak. Therefore, there is a requirement to identify novel molecular markers to improve the prognosis of gastric cancer patients.

The 14-3-3 protein family has seven distinct 14-3-3 genes, denoted β, ɛ, γ, ζ, σ, η and τ. Thus far, 14-3-3 proteins have been identified to participate in the biological regulation of cell behavior such as apoptosis, the cell cycle and malignant transformation, by interacting with ligands (8,9). Of the seven 14-3-3 genes, 14-3-3σ has been identified as an epithelial-specific marker and is associated with G2/M checkpoint control in the cell cycle (10); 14-3-3σ induced the activation of tumor protein p53, and bound to cyclin-dependent kinase-2 (CDK2) and -4 (CDK4) (11). Thus, 14-3-3σ may be defined as a negative regulator of the cell cycle. Protein expression levels of 14-3-3σ are significantly reduced or negligible in various types of primary cancer of epithelial origin, including lung (12), prostate (13) and bladder carcinoma (14). This cancer-related inactivation of the 14-3-3σ gene may be caused by hypermethylation of CpG islands in the promoter region of 14-3-3σ (15). However, to date, few studies have reported a correlation between 14-3-3σ expression levels and clinicopathological features of gastric cancer. Therefore, to investigate the clinical significance and prognostic value of 14-3-3σ in gastric cancer, the present study analyzed 14-3-3σ expression in 60 gastric cancer samples and evaluated its correlation with specific clinical outcomes of gastric cancer patients.

## Materials and methods

### Patients and tissue samples

Sixty paraffin-embedded gastric cancer samples were collected from The Central Hospital of Loudi Affiliated to the University of South China (Loudi, China), between 2007 and 2008. None of the patients had received preoperative anticancer treatment. Clinicopathological features, including age, gender, tumor differentiation degree, tumor volume, tumor invasion depth, tumor node metastasis (TNM) stage and lymph node metastasis are detailed in Table I. Informed consent was obtained from each patient upon collection of the samples. The present study was approved by the institutional research medical ethics committee of The Central Hospital of Loudi Affiliated to the University of South China.

### Tissue microarray and immunohistochemistry

Tissue microarrays (TMAs) were constructed as previously described (16). Briefly, paraffin-embedded donor tissue blocks and the corresponding hematoxylin and eosin-stained slides were overlaid for TMA sampling. Cylindrical tissue samples (diameter, 0.6 mm) were punctured in triplicate from specific areas of the donor tissue and re-embedded into a recipient paraffin block at the designated location.

Routine techniques were utilized to cut 4-μm sections from the deparaffinized TMAs. The slides were microwaved in citrate buffer for 8 min for antigen retrieval, and goat anti-human polyclonal 14-3-3σ (sc-7683; Santa Cruz Biotechnology, Inc., Santa Cruz, CA, USA) was applied as the primary antibody in a 1:150 dilution. Goat anti-human monoclonal Ki-67 (GT209401), goat anti-human polyclonal Bcl-2 (GM088701) and goat anti-human Bax (A353302) were purchased from Gene Biotechnology (Shanghai, China). Labeling was detected by horseradish peroxidase-conjugated mouse anti-goat IgG and staining with 3,3′-diaminobenzidine (all Maxim-Bio, Inc., Fuzhou, China). Finally, the slides were counterstained with hematoxylin. 14-3-3σ, Bcl-2 and Bax were scored according to the staining intensity (0, no staining; 1, weak staining; 2, moderate staining; 3, strong staining) and the percentage of positively stained tumor cells (0, 0% of tumor cells stained; 1, 1–9% of tumor cells stained; 2, 10–50% of tumor cells stained; 3, 51–75% of tumor cells stained; 4, >75% of tumor cells stained). If the product of the staining intensity and the percentage of positively stained tumor cells was ≥2, the staining was considered to be positive (+). Ki-67 was scored according to the number of positive gastric cancer cells. A negative control was performed by replacing the primary antibody with the appropriate serum controls.

### Statistical analysis

All statistical analyses were performed using SPSS for Windows (version 13.0; SPSS, Inc., Chicago, IL, USA). The correlation between 14-3-3σ expression rates and clinicopathological features of gastric cancer cases was evaluated using Fisher’s exact test. Multivariate analysis was performed using Cox proportional-hazards regression. Overall survival curves were generated according to the Kaplan-Meier method. P<0.05 was considered to indicate a statistically significant difference.

## Results

### Expression of 14-3-3σ in gastric cancer

**Figure 1:**
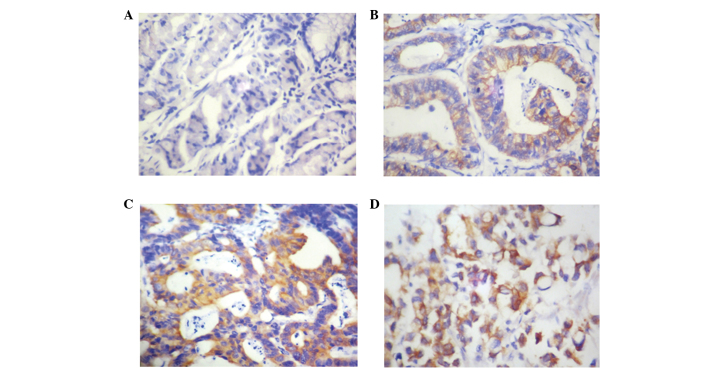
Expression pattern of 14-3-3σ in gastric cancer. (A) No 14-3-3σ expression in healthy gastric tissues. (B) Overexpression of 14-3-3σ in the cytoplasm of well-differentiated gastric cancer cells. (C) High 14-3-3σ expression in moderately differentiated gastric cancer cells. (D) Positive 14-3-3σ expression in poorly differentiated gastric cancer cells (magnification, ×200).

To investigate the expression of 14-3-3σ in gastric cancer, immunohistochemical staining for 14-3-3σ was performed on a TMA containing 60 pairs of gastric cancer samples and the corresponding healthy gastric mucosa tissues. In the present study, compared with weak or negative expression in normal gastric mucosal tissues, 63.3% (38/60) of gastric cancer cases exhibited positive 14-3-3σ expression. As demonstrated in Fig. 1, 14-3-3σ staining was evident in the cytoplasm of poorly, moderately and well-differentiated gastric cancer cells.

### Association between 14-3-3σ expression and clinicopathological features of gastric cancer

Table I indicates the expression rates of 14-3-3σ in gastric cancer with respect to various clinicopathological features. The expression rates of 14-3-3σ were significantly higher in patients exhibiting larger tumors (size, ≥5 cm; P=0.017) and an advanced TNM stage (P=0.003). However, no significant difference was identified between 14-3-3σ expression and the other clinicopathological features investigated, such as age, gender, tumor differentiation degree, depth of tumor invasion and lymph node metastasis (P>0.05).

### Association between 14-3-3σ and Ki-67 expression

To explore the influence of 14-3-3σ on the proliferation of gastric cancer cells, the present study analyzed the correlation between 14-3-3σ and Ki-67 expression. The percentage of cells exhibiting positive Ki-67 expression was significantly higher (48.1±5.1) in the 14-3-3σ positive expression group compared with the 14-3-3σ negative expression group (17.6±4.8) (Table II). These findings indicate that 14-3-3σ may participate in tumor cell proliferation.

### Association between 14-3-3σ and Bcl-2/Bax expression

**Figure 2:**
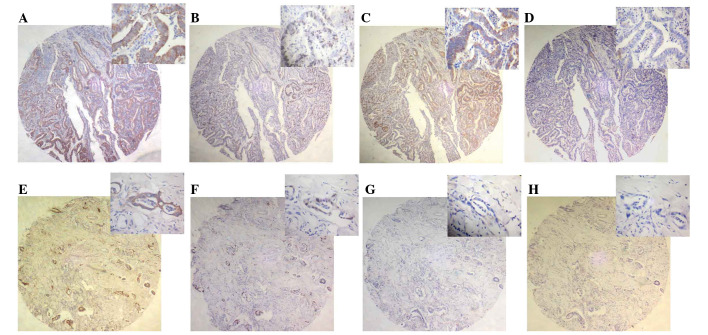
Immunohistochemical staining of 14-3-3σ, Ki-67, Bcl-2 and Bax in the gastric cancer. Case 1: (A–C) Positive expression of 14-3-3σ (A), Ki-67 (B) and Bcl-2 (C), and (D) negative expression of Bax. Case 2: (E–G) Positive expression of 14-3-3σ (E), Ki-67 (F), and Bcl-2 (G), and (H) negative expression of Bax (low power magnification, ×100; high power magnification, ×200).

To identify the effect of 14-3-3σ on the apoptosis of gastric cancer cells, the present study investigated the correlation between 14-3-3σ and Bcl-2/Bax expression. Bcl-2 staining was identified in 25/38 cases of positive 14-3-3σ expression (Table II). As demonstrated in Fig. 2, Bcl-2 expression was predominantly present within the cytoplasm of gastric cancer cells. Furthermore, 14/22 cases of negative 14-3-3σ expression did not exhibit Bcl-2 staining (Table II). However, with the exception of six positive expression cases, no Bax immunoreactivity was detected in these samples.

### Association between 14-3-3σ expression and overall survival in gastric cancer

**Figure 3:**
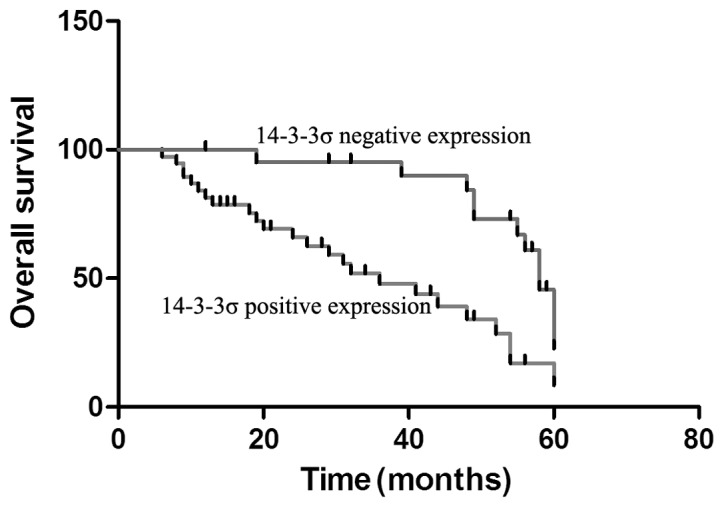
Kaplan-Meier survival analysis of 14-3-3σ expression in gastric cancer patients. Patients with negative 14-3-3σ expression exhibited a more favorable prognosis than those with positive 14-3-3σ expression.

To better elucidate the effect of 14-3-3σ expression on the prognosis of gastric cancer, Kaplan-Meier survival curves were used to investigate the overall survival time of gastric cancer patients. Negative 14-3-3σ expression patients exhibited improved prognoses compared with positive 14-3-3σ expression patients (Fig. 3). Additionally, univariate and multivariate analyses were employed to assess the role of 14-3-3σ expression and specific clinicopathological features on the prognosis of gastric cancer patients. Univariate analysis demonstrated that lymph node metastasis, TNM stage, tumor invasion depth and 14-3-3σ expression were significantly associated with the overall survival of gastric cancer patients. Furthermore, multivariate analyses identified that lymph node metastasis, TNM stage and 14-3-3σ expression were correlated with poor overall survival of gastric cancer patients (Table III). Of the clinicopathological features investigated in the present study, lymph node metastasis was the most independent prognostic indicator of gastric cancer (P=0.013). Therefore, 14-3-3σ expression may act as a prognostic biomarker for gastric cancer.

## Discussion

14-3-3σ is vital at the G2/M checkpoint, as it sequesters the Cdc2/cyclin B1 complex; therefore, 14-3-3σ may be involved in the development of cancer. However, it should be considered that 14-3-3σ exhibits different functions in the carcinogenesis of different human organs. For example, downregulation of 14-3-3σ expression has been observed in colon (17), liver (18), breast (19) and ovarian cancer (20), and loss of 14-3-3σ expression has been associated with poor prognosis in epithelial ovarian carcinoma (20). By contrast, 14-3-3σ expression is upregulated in head and neck squamous cell carcinoma (21) and pancreatic cancer (22). This data supports a dual role for 14-3-3σ in the development of cancer.

In the present study, 14-3-3σ expression was significantly higher in gastric cancer compared with corresponding healthy gastric tissue. Immunohistochemical assays demonstrated that cases of gastric cancer exhibiting high 14-3-3σ expression levels also exhibited larger tumor volumes, indicating that 14-3-3σ may be involved in gastric cancer proliferation. Furthermore, 14-3-3σ expression was correlated with TNM stage. This finding was consistent with those of a previous study conducted by Perathoner *et al* (23), which revealed a significant correlation between 14-3-3σ overexpression and tumor stage in colorectal carcinoma. These characteristics of 14-3-3σ expression may influence the prognosis of gastric cancer patients. With regard to the prognostic impact of 14-3-3σ in human cancer, positive 14-3-3σ overexpression was associated with a significantly decreased survival time compared with negative 14-3-3σ expression in colorectal carcinoma (23). Consistent with this, the present study performed Kaplan-Meier survival analysis and multivariate Cox proportional-hazards regression analysis, which revealed that a high expression of 14-3-3σ was significantly associated with a poorer prognosis and shortened overall survival time.

In the present study, 14-3-3σ expression was correlated with tumor volume. Therefore, the association between 14-3-3σ, and proliferation and apoptosis in gastric cancer was investigated. The results of the present study demonstrated a positive correlation between 14-3-3σ and Ki-67, indicating that 14-3-3σ may be associated with the proliferation of gastric cancer cells. 14-3-3σ exerts anti-apoptotic effects by interacting with Bax, a pro-apoptotic protein (24). To investigate the role of 14-3-3σ in the apoptosis of gastric cancer cells, the correlation between 14-3-3σ and Bcl-2/Bax was explored. The positive rate of Bcl-2 expression in the 14-3-3σ-positive cases was significantly higher compared with the rate of Bcl-2 expression in the 14-3-3σ-negative cases. Furthermore, no correlation was identified between 14-3-3σ and Bax expression.

In conclusion, the present study investigated the expression pattern of 14-3-3σ in gastric cancer and its correlation with specific clinicopathological features. The results of the present study indicate that 14-3-3σ may be involved in cell proliferation and apoptosis in gastric cancer. Furthermore, 14-3-3σ overexpression is associated with an unfavorable prognosis. Therefore, 14-3-3σ may be a promising target for the treatment of gastric cancer and, critically, investigations into the 14-3-3σ-associated molecular mechanisms of gastric cancer cell proliferation and apoptosis should be conducted.

## Figures and Tables

**Table I:** Correlation between 14-3-3σ expression rate and various clinicopathological features in 60 cases of gastric cancer.

		14-3-3σ expression		
			
Variable	Cases, n	Negative	Positive	P-value
Gender				
Male	31	15	16	0.064
Female	29	7	22	
Age, years				
≥60	28	11	17	0.791
<60	32	11	21	
Tumor size, cm				
≥5	26	5	21	0.017^a^
<5	34	17	17	
Differentiation degree				
Well/Moderately	39	13	26	0.577
Poorly	21	9	12	
Invasion depth				
T1 + T2	41	18	23	0.149
T3 + T4	19	4	15	
TNM stage				
I + II	34	18	16	0.003^a^
III + IV	26	4	22	
Lymph node metastasis				
Yes	37	17	20	0.097
No	23	5	18	

a P<0.05.

TNM, tumor node metastasis.

**Table II:** Correlation between 14-3-3σ, Bcl-2 and Ki-67 expression.

		Bcl-2 staining		
			
14-3-3σ staining	Cases, n	Positive	Negative	Ki-67, % (±SD)
Positive	38	25^a^	13	48.1±5.1^a^
Negative	22	8	14	17.6±4.8

a P<0.05, vs. negative 14-3-3σ staining.

SD, standard deviation.

**Table III:** Univariate and multivariate analyses of overall survival in gastric cancer patients.

	Univariate analysis			Multivariate analysis		
		
Variable	Hazard ratio	95% CI	P-value	Hazard ratio	95% CI	P-value
Gender	1.561	0.832–3.914	0.372			
Age, year	1.126	0.982–1.145	0.541			
Invasion depth	5.530	2.479–13.885	0.001^a^	2.726	0.789–6.543	0.054
Differentiation degree	0.986	0.323–2.879	0.788			
Tumor size	1.013	0.346–3.190	0.977			
TNM stage	0.104	0.032–0.501	0.003^a^	0.195	0.040–0.765	0.026^a^
Lymph node metastasis	6.114	3.062–18.767	0.002^a^	3.378	1.483–9.323	0.013^a^
14-3-3σ expression	5.983	2.690–17.654	0.002^a^	3.896	1.719–9.891	0.028^a^

a P<0.05.

CI, confidence interval; TNM, tumor node metastasis.
